# Rapid detection of drug resistance in *Mycobacterium tuberculosis* clinical isolates for first-line antitubercular drugs by using a novel reporter mycobacteriophage

**DOI:** 10.3389/fcimb.2025.1589236

**Published:** 2025-04-10

**Authors:** Mingquan Guo, Yan Wang, Juntao Sun, Chengcheng Qian, Douglas B. Lowrie, Liangfei Niu, Juan Wu, Zhidong Hu, Xiao-Yong Fan, Ruiqing Ma

**Affiliations:** ^1^ Shanghai Public Health Clinical Center & Shanghai Institute of Infectious Diseases and Biosecurity, Fudan University, Shanghai, China; ^2^ Department of Anesthesiology, Renji Hospital, Shanghai Jiao Tong University School of Medicine, Shanghai, China; ^3^ Department of Gastroenterology, The Second Hospital, Cheeloo College of Medicine, Shandong University, Jinan, Shandong, China; ^4^ Department of Laboratory Medicine, Affiliated Hospital of Nantong University, Nantong, Jiangsu, China

**Keywords:** *Mycobacterium tuberculosis*, reporter phage &phiv;FN, drug susceptibility testing (DST), first-line antitubercular drugs, drug resistance

## Abstract

The emergence of drug resistance presents a major challenge for the treatment of tuberculosis (TB). Reporter phage can provide an effective method for drug susceptibility testing (DST) of *Mycobacterium tuberculosis* (*Mtb)*, but limited for parts of antitubercular drugs and the process of operation is usually time-consuming. Herein, we developed a new, sensitive, reporter phage with optimized method to detect drug susceptibility of *Mtb* clinical isolates for all first-line antitubercular drugs. The P*_furAma_* promoter and nanoluciferase (Nluc) reporter sequences were integrated into the genome of the TM4 mycobacteriophage to generate a reporter phage, designated φFN. By optimizing concentration of *Mtb*, Tween 80 and drugs, we have established an efficient workflow for φFN-based DST of *Mtb* that provides results for four first-line antitubercular drugs within 72 hours. A total of 71 clinical isolates were tested and yielded significant relative luminescent units (RLUs), and their resistance to rifampin (RIF), isoniazid (INH), streptomycin (STR), and EMB were compared to the conventional DST by MGIT 960. The comparative sensitivities of φFN DST detection were 100%, 93.9%, 97.2%, and 81.3%, respectively; and the relative specificities were 98.1%, 97.4%, 97.1%, and 96.4%, respectively. The remaining luminescence rate (RLR) in the φFN DST assay showed correlation with minimum inhibitory concentration (MIC). The φFN DST assay provides an efficient phage-based workflow to detect drug-resistant *Mtb* for four first-line antitubercular drugs within 3 days.

## Introduction

Tuberculosis (TB) is caused by the intracellular pathogen *Mycobacterium tuberculosis* (*Mtb*). In 2023, approximately 10.8 million new cases of TB were diagnosed globally, with an estimated 1.25 million deaths. *Mtb* is the second leading cause of mortality by a single pathogen worldwide, following COVID-19 (World Health Organization, 2024). Although antibiotics such as rifampicin (RIF) can effectively cure the disease, the emergence of drug-resistant forms of *Mtb* (DR-TB), such as Rif-resistant TB (RR-TB), multidrug-resistant TB (MDR-TB), and extensively drug-resistant TB (XDR-TB), has resulted in a public health crisis in which TB has a low cure rate, and lengthy and costly treatment cycles (Farhat et al., 2024).

Identification of drug resistance is a crucial aspect of treating DR-TB. This can be challenging due to the slow-growing nature of *Mtb*, particularly when there are low bacterial loads of TB in clinical samples, and unknown drug-resistance mechanisms involved. In areas with a high incidence of TB, the proportion method with solid media is usually used to test susceptibility to most first- and second-line TB drugs. This method is currently the “gold standard” for drug resistance detection, but most of the commonly used phenotypic DSTs require pure cultures of MTB, which takes a long time to develop (> 30 days for culture positivity) (World Health Organization, 2018). Although a number of genotype-based, rapid molecular DST technologies have been developed, such as GeneXpert MTB/RIF, next-generation sequencing, gene chips, and CRISPR assay, resistance to only a few drugs is currently detectable by these methods due to a lack of understanding of the underlying drug-resistance mechanisms (Xiong et al., 2024).

For decades, mycobacteriophages have been employed in the development of DST methods for *Mtb*, because they are comparatively low-cost, rapid, and universally applicable for antitubercular drugs. The first reporter mycobacteriophage for DST used an *hsp60* promotor and firefly luciferase gene integrated into the genome of mycobacteriophage TM4 and was demonstrated to be applicable for DST of RIF and INH (Jacobs et al., 1993). φ^2^GFP10 was later constructed by expressing GFP under a powerful promoter, P*_left_*, and exhibited high sensitivity and specificity in DST of clinical isolates for INH, RIF, and STR (Jain et al., 2012; Yu et al., 2016). Further modifications were implemented to enhance the expression of reporter gene cassettes, thereby highlighting the potential of mycobacteriophages in DST of *Mtb* (Jain et al., 2016; Urdániz et al., 2016). One such modification (TM4-nluc), utilizing *nluc* (nanoluciferase) as a reporter gene, was shown to detect low bacterial loads (limit of detection ≤ 100 CFU) and multiple drug resistances of H37Rv mc^2^6230 in 96-well plate assays (Jain et al., 2020). Recently, green-enhanced nanoluciferase (GeNL) was employed in TM4::*GeNL*, which was more sensitive than TM4-nluc and performed well in DST of *Mtb* for resistance to bedaquiline and RIF (Rajagopalan et al., 2024).

*Mtb* DST by reporter phage is a well-established method, but none of the current phages have been reported to identify drug-resistance *Mtb* for all first-line antitubercular drugs, especially for clinical isolates. Additionally, cumbersome procedures present a significant challenge for the detection of resistance in numerous clinical samples against multiple drugs. Here, we report the construction of a highly-sensitive reporter phage, φFN, for *Mtb*. By optimizing the process of operation, we have developed an efficient, rapid, and sensitive, φFN DST assay for 4 first-line antitubercular drugs, RIF, isoniazid (INH), streptomycin (STR), and ethambutol (EMB) within 3 days only.

## Materials and methods

### Bacterial strains and culture conditions

All clinical strains were isolated from TB patients at Shanghai Public Health Clinical Center (Shanghai, China) and conventional DST was performed by MGIT 960 in clinical laboratory. *Mycobacterium smegmatis* mc^2^155, *Mycobacterium tuberculosis* H37Rv, and clinical strains were grown, routinely, at 37°C in liquid Middlebrook 7H9 broth (BD Difco, USA) supplemented with 10% (v/v) oleic acid-albumin-dextrose-catalase enrichment (OADC; BD Difco, USA), 0.5% glycerol, and 0.05% Tween 80. 7H11 agar supplemented with 10% OADC, 0.5% glycerol was used as a solid culture medium.

### Expression of reporter cassettes in *Mycobacterium smegmatis*

The P*_furAma_* promoter was cloned from plasmid pMFA41 (Fan et al., 2009). Promoter P*_left_* (Nesbit et al., 1995) was synthesized by Genewiz, and *nluc* was provided by Promega (Wisconsin, USA). The *nluc* and promoters were assembled into pMV361 (Stover et al., 1991) using the Gibson assembly method (Gibson et al., 2009). Plasmids were electroporated into *Mycobacterium smegmatis* mc^2^155 to achieve integrative expression of the reporter cassettes.

### Construction of φFN phage

The plasmid pYUB854, and TM4-derived phage backbone phAE159, were donated by Dr William R. Jacobs’ laboratory at Albert Einstein University (New York, USA) (Bardarov et al., 2002). Reporter cassette P*_furAma_*-*nluc* was cloned into pYUB854 using the Gibson assembly method (Gibson et al., 2009). The resulting plasmid was then digested with PacI and introduced into phAE159 using the MaxPlax *in vitro* packaging extract (Epicentre) to generate shuttle phasmids. A total of 500 ng shuttle phasmid was electroporated into 300 μL *Mycobacterium smegmatis* mc^2^155 (*Msm*) competent cells, and the cells were recovered after adding 1 mL 7H9 medium without Tween 80 for 1 hour at 37°C. A 300 μL volume of recovered cells was mixed with 10 mL of top agar (0.7%) and poured over 7H11 plates. The φFN phage was harvested after 3 days of incubation at 30°C.

### Amplification and purification of phage

The amplification of phage was performed as previously described (Jain et al., 2014). Briefly, plaques were picked and added to 200 μL MP buffer (50 mM Tris-HCl, 150 mM NaCl, 10 mM MgSO_4_, 2 mM CaCl_2_, pH = 7.5), diluted to an appropriate titer, and used to infect another 300 μL of *Msm* at 30°C to obtain high titer φFN phage. The background nanoluciferase protein was reduced by ultrafiltration as previously described (Jain et al., 2020). φFN phage was layered over a CsCl solution (1.54 g/mL) and centrifuged for 22 hours at 28,000 rpm (96,281 × *g*) at 4°C. Following centrifugation, the visible middle layer fraction was collected with an 18-gauge needle and provided the purified φFN phage used in further assays.

### Measurement of relative luminescent unit

The Nano-Glo substrate (Promega, USA) was diluted 50-fold in Nano-Glo buffer and mixed 1:1 (vol:vol) with samples, in accordance with the Nano-Glo luciferase assay system manual. The mixture reacted in the wells of a 96-well flat-bottom plate, and the RLU was read immediately on a BioTek Synergy H1 plate reader with 100 gain, 0.1 s read time, and 1 mm read height.

### φFN DST assay

A 10 μL solution of precultured *Mtb*, with an OD_600_ > 0.2, was added to 1 mL 7H9 media without Tween 80 and mixed by vortex; 10 μL of the mixture was then added to each of the wells, containing 90 μL of 7H9 medium and one of the first-line antitubercular drugs, in 96-well plates. Control wells contained no drug. The final concentrations of RIF, INH, STR, and EMB were 1 mg/L, 0.1 mg/L, 1 mg/L and 1 mg/L respectively. The concentrations of RIF, INH, and STR were those recommended by WHO for the MGIT DST. Wells containing 7H9 and drugs without *Mtb* were used as blanks. After 48 hours incubation, 10 μL of 10^8^ pfu/mL φFN phage was added to the wells, which were incubated for a further 24 hours. The RLU was measured as described above, and the RLR value was calculated using the formula: (reaction-blank)/(control–blank) × 100%, where “reaction” represents the RLU value of a test well with drug exposure; “blank” indicates the RLU value of the well without *Mtb*; and “control” represents the RLU value of the well without drug exposure.

## Results

### Construction and evaluation of a new *M. tuberculosis* reporter phage φFN

**Figure 1:**
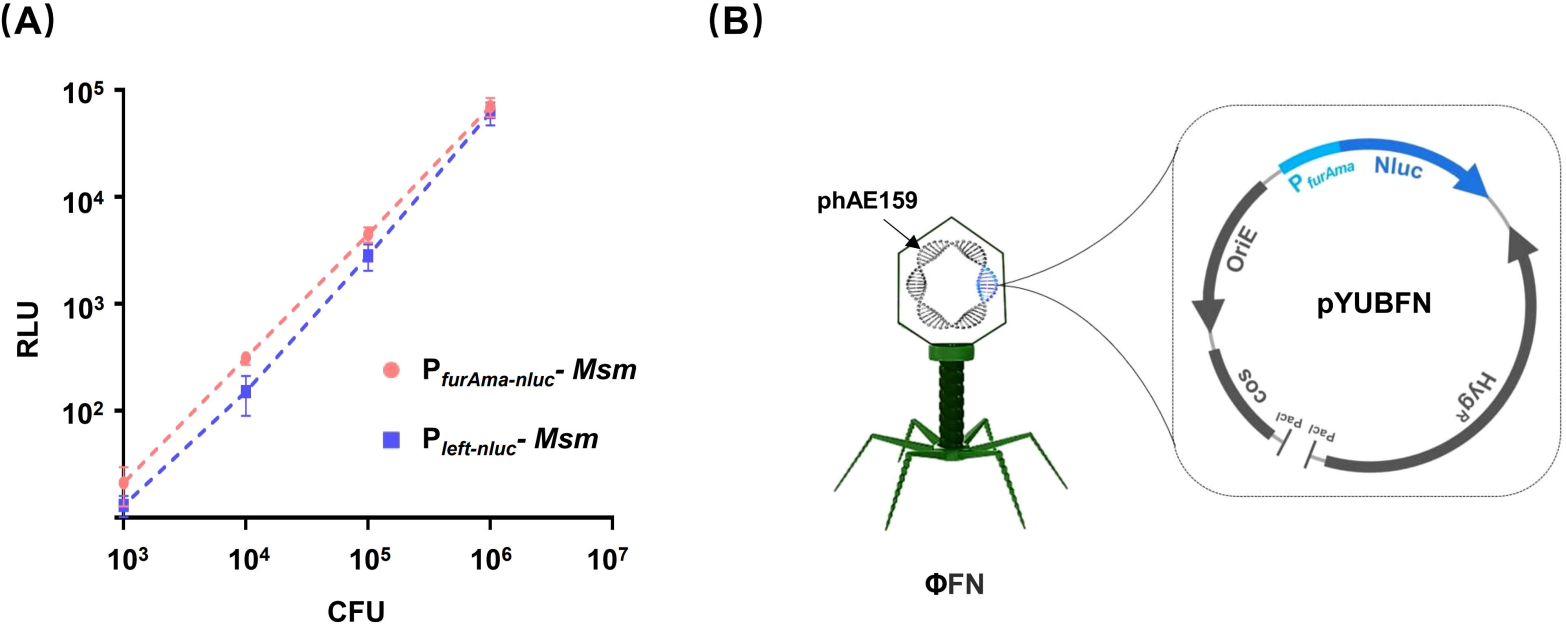
Construction of *M. tuberculosis* reporter phage. **(A)** Comparison of P*_furAma_-nluc* and P*_left_-nluc*. The reporter cassettes P*_furAma_-nluc* and P*_left_-nluc* were integrated into the genome of *Mycobacterium smegmatis* mc^2^155 (*Msm*). Precultured *Msm* were washed twice with PBS before 10-fold serial dilution in 96-well plates. Luminescence and CFU were assessed in parallel series dilutions. The Y-axis shows the relative luminescence units (RLU) against the bacterial load (X-axis) as determined by spread plate on 7H11 agar. **(B)** Schematics of the recombinant Mycobacteriophage φFN. The φFN genome consisted of a phAE159 backbone, and pYUBFN containing the P*_furAma-nluc_* reporter gene cassette.

As Nluc has been shown to exhibit superior luciferase reporting capability (Jain et al., 2020; England et al., 2016), we cloned the Nluc encoding gene (*nluc*) downstream of the constitutively expressed promoter P*_furAma_* to assemble an *Mtb* reporter cassette (Fan et al., 2009). When expressed in *Mycobacterium smegmatis*, the P*_furAma_-nluc* reporter cassette exhibited similar RLU to P*_left_-nluc* (**Figure 1A**). Accordingly, the reporter cassette was subsequently integrated into the genome of a temperature-sensitive mycobacteriophage phAE159 (TM4) to generate a new phage, φFN (**Figure 1B**).

### DST of *Mtb* for INH, at different bacterial loads, using φFN

**Figure 2:**
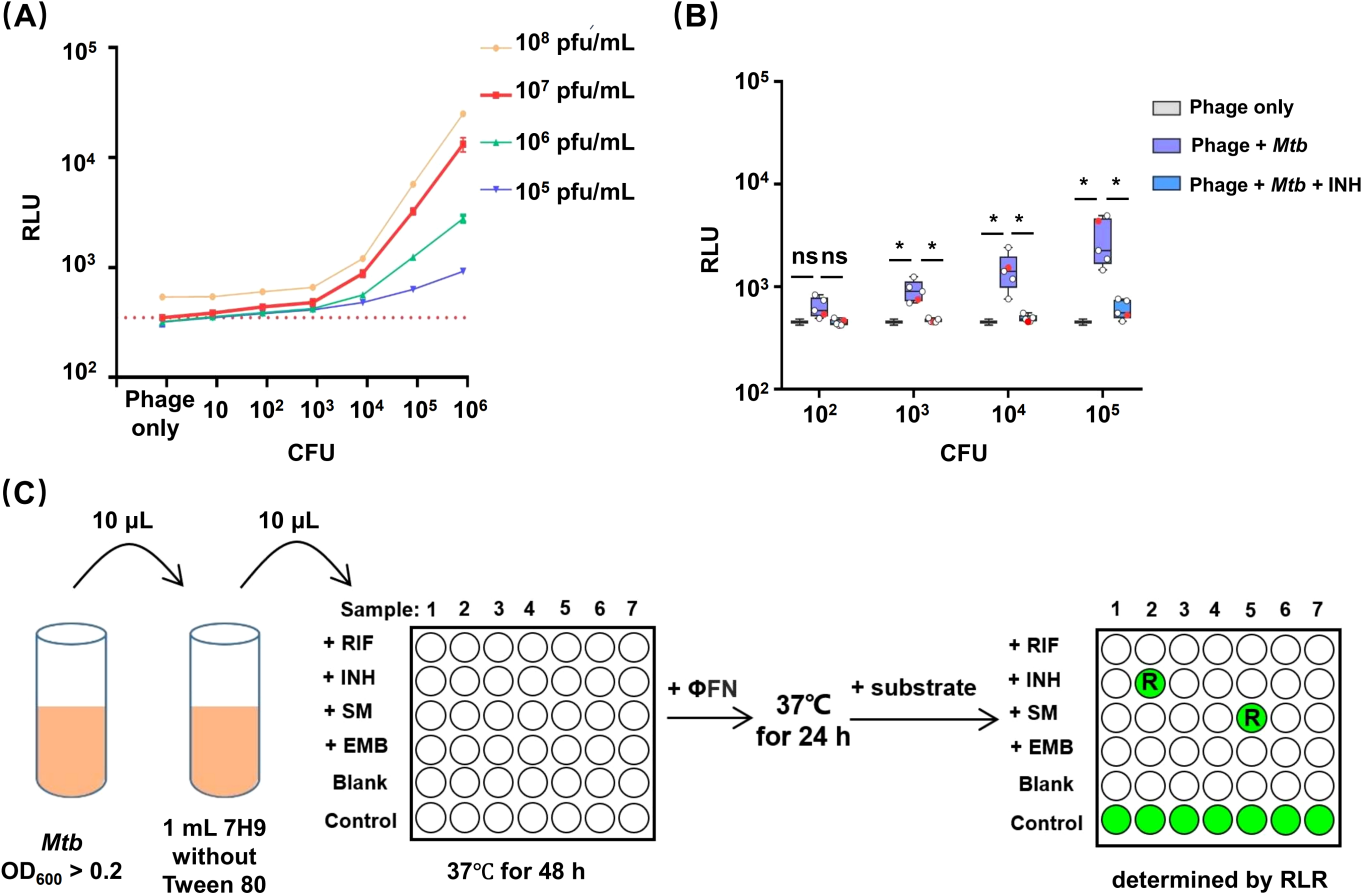
Establishment of an efficient DST workflow for using φFN. **(A)** Dependance of luminescence upon bacterial concentration and phage dose. Ten-fold serial dilutions of precultured H37Rv were incubated with different concentration of φFN in 96-well plates for 24-hour at 37°C. The red dashed line represents the background RLU of 10^6^ pfu/mL φFN. **(B)** Detection of INH resistance in clinical isolates of *Mtb* at different bacterial loads. Ten-fold serial dilutions of four INH-sensitive clinical isolates and H37Rv, from initial OD_600_ >0.2, were incubated at 37°C with, or without, INH for 48 hours, then incubated with φFN for a further 24 hours. Parallel bacterial dilutions with no drugs added were used to determine the initial CFU by spread plate on 7H11 agar. Red dots represent H37Rv. *represents *p <* 0.05 by Student’s *t-*test. **(C)** φFN DST assay workflow. Precultured *Mtb* with OD_600_ > 0.2 is diluted 100-fold in detergent-free 7H9 medium. An additional 10-fold dilution is performed in 90 μL detergent-free 7H9 ± antitubercular drugs per well in a 96-well plate. After 48 hours of incubation at 37°C, φFN is added to the wells, and incubated at 37°C for another 24 hours. Finally, Nano-Glo substrate is added, and the plate is immediately read with a luminescence detector. The blank wells have no *Mtb*. Control wells have no drug added. Drug resistance is determined from the remaining luminescence rate (RLR).

To date, studies demonstrating the ability of phages to detect the resistance of *Mtb* to RIF, INH, and STR have all used high bacterial concentrations (> 10^5^ CFU) of *Mtb*. For INH-susceptible strains, luminescence signals were still produced after exposure of INH due to slow bactericidal activities (Yu et al., 2016; Jain et al., 2020). Therefore, we tried to change the concentrations of bacterial and phage to reduce the produced luminescence signals. Precultured *Mtb* H37Rv was serially diluted and incubated with different concentrations of φFN in 96-well plates for 24 hours at 37°C, while parallel aliquots of bacteria were plated on 7H11 agar for CFU enumeration (**Figure 2A**). When CFU of *Mtb* was ≥ 10^2^, the relative luminescent units (RLU) increased with higher phage doses; a concentration of 10^7^ pfu/mL (plaque forming unit/mL) gave low background RLUs in phage-only wells and yielded the highest RLUs in the presence of *Mtb.* These results suggested that a consistent dose of phage (10^7^ pfu/mL) could be applied to detect *Mtb* in 96-well plates without consideration of bacterial concentration.

We next used φFN to test the INH resistance of H37Rv, and four INH-susceptible clinical isolates, at different bacterial loads from 10^2^ to 10^5^ CFU. The duration of drug exposure were based on previous studies (Yu et al., 2016). At *Mtb* CFUs of ≥ 10^3^, the luminescence signals produced in the wells without INH exposure were significantly higher than in the phage background, and INH exposure wells (**Figure 2B**). Additionally, luminescence could be increased by prolonging incubation with φFN from 24 to 48 hours, but a minimum CFU of 10^3^ was still required to produce significant luminescence signals (**Supplementary Figure S1**). However, when the CFU of *Mtb* was ≥ 10^5^, luminescence signals were produced in some strains of *Mtb*. These results demonstrated that low loads (10^3^~10^5^ CFU) *Mtb* were more appropriate for DST with the φFN reporter phage.

### Establishment of an efficient DST workflow, based on φFN, for low bacterial loads of *Mtb*

Before assay, precultured *Mtb* is usually washed twice by 7H9 without Tween 80 detergent, and bacterial concentrations are determined by turbidity assays in previous studies (Yu et al., 2016; Jain et al., 2020; Rajagopalan et al., 2024). The procedures may introduce inaccuracies and are time-consuming, when performed on numerous clinical isolates. Phage is unable to infect mycobacteria in conventional 7H9 medium containing 0.1% Tween 80. However, the ability of the phage to infect *Mtb* was restored, when the concentration of Tween 80 was diluted over 1000-fold in 7H9 (**Supplementary Figure S2**).

We accordingly optimized the procedure and developed an efficient DST workflow with φFN in which it is not necessary to consider bacterial loads, detergent effects, and the speed of action of drugs (**Figure 2C**), and named it φFN DST assay. Provided that the OD_600_ value of cultured *Mtb* is over 0.2, which can be judged by eye, the culture can be diluted with detergent-free 7H9 and exposed to drugs. After subsequent incubation with φFN, RLU is measured and the drug susceptibility of *Mtb* is determined by the remaining luminescence rate (RLR). In the process, all operations can be executed in a few minutes — without centrifugation, measurement of turbidity, or serial dilution of drugs — and the DST of multiple samples can be performed in a single batch.

### Detection of *Mtb* ethambutol resistance by the φFN DST assay

**Figure 3:**
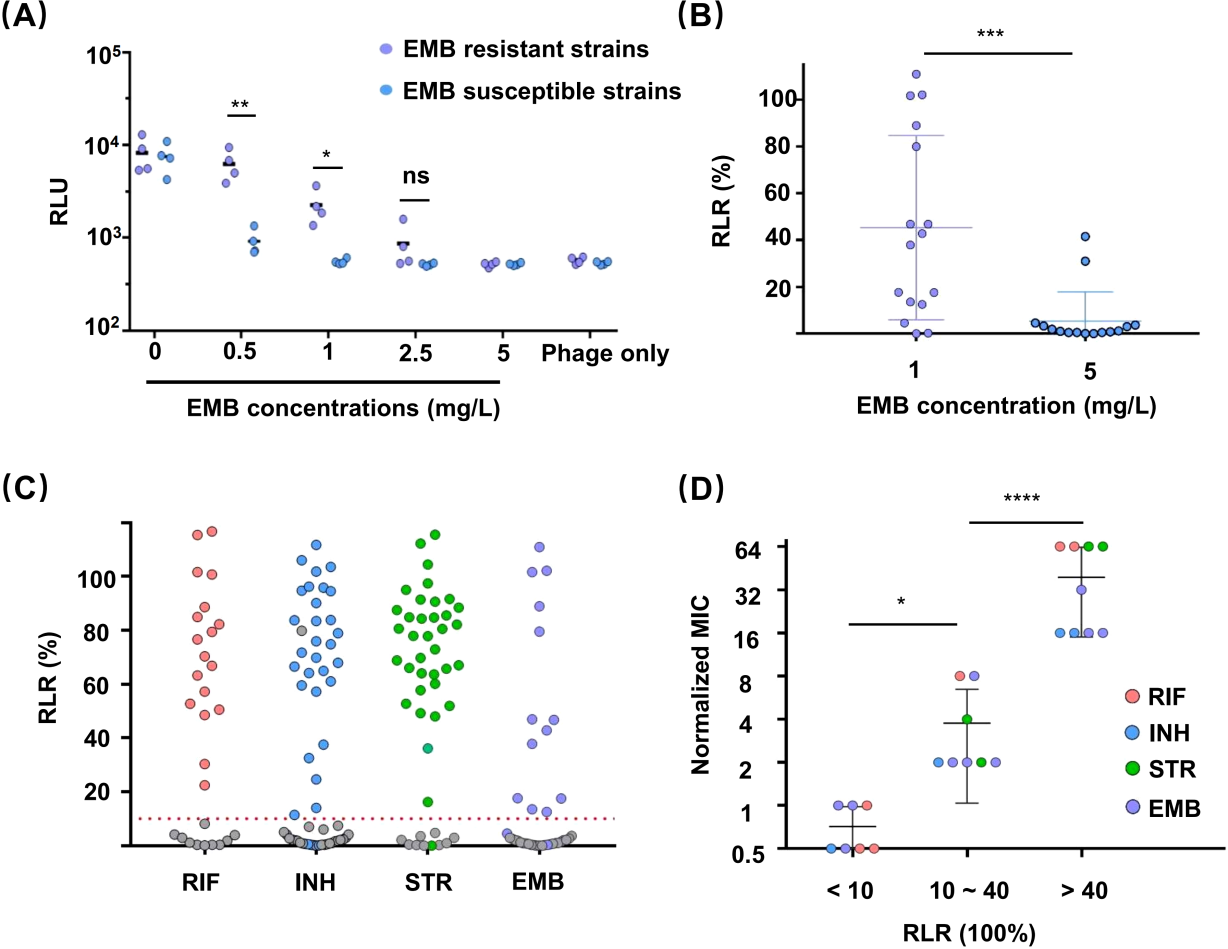
Drug susceptibility testing (DST) of clinical isolates for first-line antitubercular drugs using the φFN assay. The assays were performed as described in **Figure 2C**. **(A)** Resistance of clinical isolates to EMB. EMB-sensitive and -resistant clinical isolates were exposed to different concentrations of EMB for 48 hours, then incubated with φFN for a further 24 hours before luminescence assay and calculation of RLU. *represents *p <* 0.05, and **represents *p <* 0.01, based on Student’s *t-*test. **(B)** φFN DST assays confirming the difference between exposure to 1 mg/L and 5 mg/L EMB. Fifteen clinical isolates were analyzed. ***represents *p <* 0.001, based on Student’s *t-*test. **(C)** Comparison of MGIT 960 and φFN DST assays for the detection of drug resistance of clinical isolates to first-line antitubercular drugs. The RLR of 71 clinical isolates, for which resistance or susceptibility to first-line antitubercular drugs was previously established by MGIT 960 testing, were assessed by φFN DST assay. Colored and gray dots represent resistant and sensitive strains, respectively, as identified by MGIT 960. The red dashed line indicates RLR = 10%. **(D)** Correlation between RLR and the degree of drug resistance. The MICs of 24 isolates were established by standard clinical MIC assays. The normalized MIC was the ratio of MIC and drug concentration used in φFN DST assays. *represents *p <* 0.05, and ****represents *p* < 0.0001, based on Student’s *t-*test.

Previous studies have demonstrated the applicability of reporter phages to the detection of resistance to RIF, INH, and STR, but not to EMB (Yu et al., 2016). To assess the applicability of this new method to all four first-line antitubercular drugs, four EMB-susceptible and four EMB-resistant clinical isolates were tested in the φFN DST assay. The isolates were exposed to the concentration of EMB (5 mg/L) specified by the WHO for use in the MGIT 960 method (World Health Organization, 2018), but none of the resistant clinical isolates produced luminescence signals (**Figure 3A**). Therefore, a range of concentrations of EMB were subsequently tested, and a discernible difference between the resistant and susceptible strains emerged when the EMB concentration was less than 1 mg/L. Since luminescence signals were produced in susceptible strains at an EMB concentration of 0.5 mg/L, we ultimately chose 1 mg/L EMB as the concentration for routine use and performed φFN DST assays on 15 further EMB-resistant clinical isolates. The results showed that the detection rate of resistant strains was markedly enhanced at 1mg/L EMB, in comparison to 5 mg/L (**Figure 3B**).

### Relationship between RLR and drug resistance

Different clinical isolates with resistance to the same drug can exhibit varying degrees of resistance because of genotype (Liu et al., 2018). For devising TB treatment strategies, it is valuable to define the drug resistance level of *Mtb*, such as minimum inhibitory concentration (MIC), which cannot be determined by MGIT 960. A recent study found that the RLR might not serve as a potential indicator of MIC for RIF (Rajagopalan et al., 2024). Therefore, we tried to investigated whether the RLR, as determined by φFN DST assay, could be indicative of the MIC. Twenty-four clinical isolates with resistance to different drugs were divided into three groups according to their RLR (< 10%, 10% ~ 40%, and >40%), and were all assayed for their respective MICs (**Figure 3D**). The *Mtb* in the medium RLR group (10% ~ 40%) exhibited a degree of drug resistance that was significantly lower than that of the high RLR group (>40%), which suggests that the value of RLR obtained by φFN DST assay could be indicative of the level of drug resistance of *Mtb* to these first-line antitubercular drugs.

### DST of clinical isolates with four first-line antitubercular drugs using the φFN DST assay

**Figure 4:**
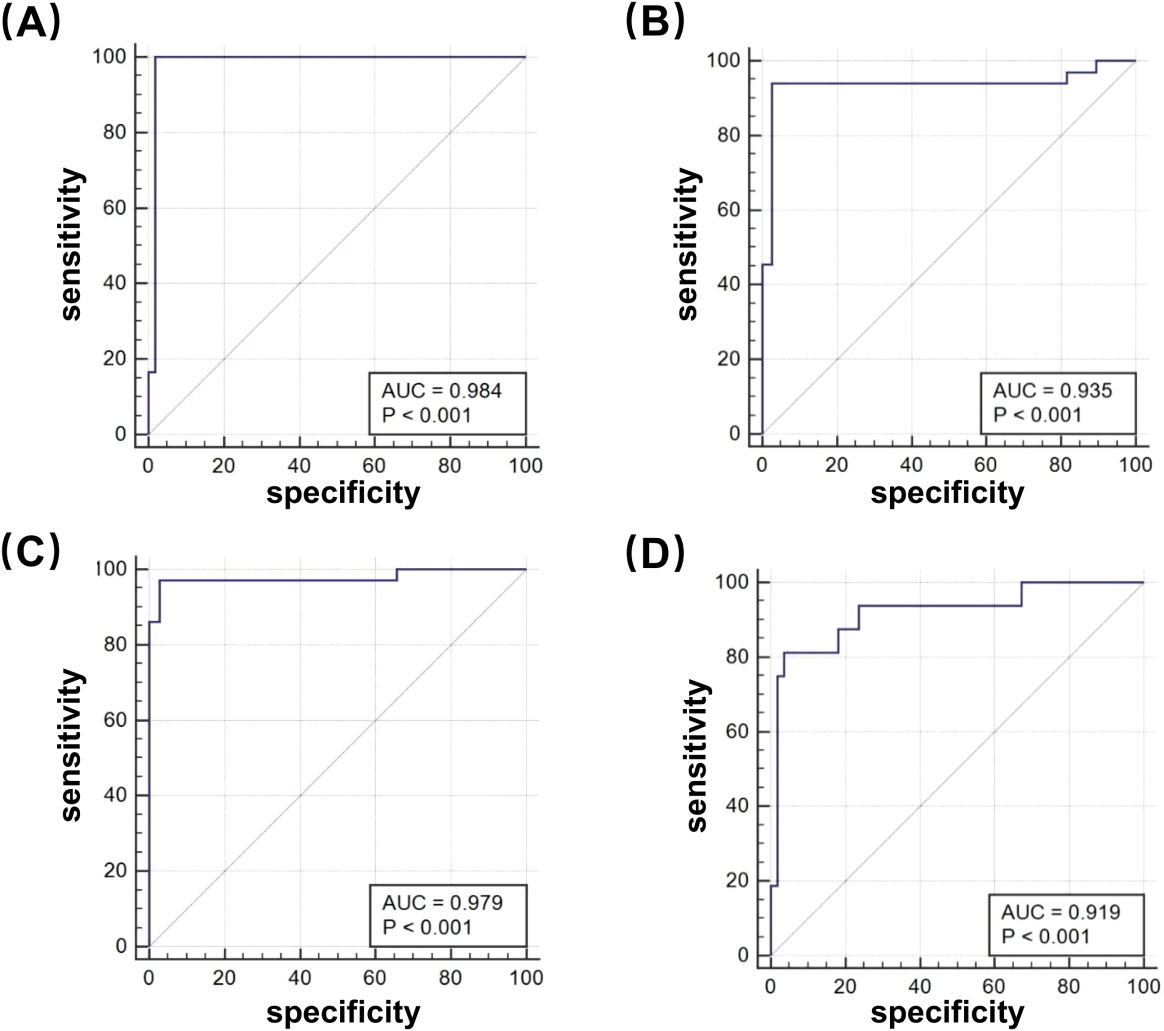
Receiver operating characteristic (ROC) curves of the φFN DST assay. The φFN DST assay was compared with the MGIT 960 system, for the detection of drug-resistance to first-line antitubercular drugs: **(A)** RIF; **(B)** INH; **(C)** STR; and **(D)** EMB.

To evaluate the effectiveness of the φFN DST assay, a total of 71 clinical isolates with resistance to first-line antitubercular drugs were tested by φFN. Significant RLUs were produced and the RLRs were calculated (**Figure 3C**). In comparison to the outcomes of the original MGIT 960 testing, the relative sensitivity of the φFN DST assay for RIF, INH, STR, and EMB was 100%, 93.9%, 97.2%, and 81.3%, respectively, and the specificity was 98.1%, 97.4%, 97.1%, and 96.4% (**Table 1**). The area under the curve (AUC) in the ROC curves for RIF, INH, STR, and EMB were 0.98, 0.94, 0.98, and 0.92, respectively (**Figure 4**). These results demonstrated that the φFN DST assay is applicable and accurate in the detection of resistance of *Mtb* to these four first-line antitubercular drugs.

**Table 1:** Performance of φFN DST assay compared to MGIT 960 in detection of drug resistance to first-line antitubercular drugs in 71 clinical isolates.

Antibiotic	AUC	RLR Cutoff (%)	% (95% Cl)		φFN DST assay		MGIT 960	
			Sensitivity	Specificity	R^a^	S^b^	R	S
RIF	0.98	8.1	100.0 (81.5 - 100.0)	98.1 (89.9 - 100)	19	52	19	52
INH	0.94	7.5	93.9 (79.8 - 99.3)	97.4 (82.6 - 99.9)	31	40	33	38
STR	0.98	4.7	97.2 (85.5 - 99.9)	97.1 (85.1 - 99.9)	35	36	36	35
EMB	0.92	3.6	81.3 (54.5 - 96.0)	96.4 (87.5 - 99.6)	16	55	16	55

^a^resistant; ^b^sensitive.

## Discussion

TB remains a serious public health problem globally, and MDR-TB and XDR-TB are current clinical challenges that need to be addressed. Definitive antibiotic susceptibility testing is the key to clinical treatment and epidemic prevention and control. It has been over 30 years since modified mycobacteriophages were first used to detect *Mtb* and its drug resistance. Some studies have demonstrated that phage-based methods for detecting drug resistance of *Mtb* are low-cost, rapid, and accurate. However, obstacles have persisted for the application of phages to DST of clinical samples, including cumbersome processes, such as quantification of bacterial load and phage dose, dilution of drugs, and removal of Tween 80; and ambiguity in the determination of drug resistance due to the varied bacteriostatic/bactericidal activities of the drugs tested (Yu et al., 2016; Urdániz et al., 2016; Jain et al., 2020; Rajagopalan et al., 2024). Recently, TM4::*GeNL* exhibited limits of detection (LOD) of ≤ 10^2^ CFU for H37Rv mc^2^6230 and available to DST of clinical isolates for bedaquiline and RIF, but the LOD for H37Rv mc^2^6230 showed significant difference with clinical isolates and the drug resistance of several samples determined by TM4::*GeNL* could not match with MGIT DST for RIF (Rajagopalan et al., 2024). Thus, more convenient and general methods still need to be developed.

In this work, we integrated a new reporter cassette P*_furAma_*-nluc into genome of phAE159 to generate a new phage, φFN. We used a range of bacterial and phage concentrations to define optimal phage concentration and 10^7^ pfu/mL showed significant generation of luminescence signals, low background, and high usability for *Mtb*concentrations of 10^2^ to 10^6^ CFU (**Figure 2A**). Therefore, it was not necessary to account for the multiplicity of infection (MOI) in the detection process. In addition, since the RLUs produced by different strains of *Mtb* after infection with phage differed significantly, it’s more appropriate to use several clinical strains of *Mtb* to evaluate the detection ability of reporter phages.

Luminescence signals were reported to still be produced after exposure of high bacterial loads (> 10^5^ CFU) of drug-susceptible *Mtb* to INH because of the poor bactericidal ability of the drug (Yu et al., 2016; Jain et al., 2020). However, our φFN assays showed effective detection ability to low bacterial loads (10^2^ ~ 10^5^ CFU) of *Mtb*; almost no luminescence signal was produced from INH-susceptible *Mtb* after incubating with INH and φFN (**Figure 2B**). Moreover, the sensitivity of DST assay for EMB was significant increased by changing the concentration of EMB from 5 mg/L to 1 mg/L (**Figure 3A**), which was reported for the first time on the utilization of phage to identify EMB drug resistance of *Mtb*. However, further investigation is required to improve sensitivity for EMB (81.3%) and ascertain the potential of φFN for the detection of second-line antitubercular drugs.

With clinical isolates, the estimation of CFU by turbidity assays in liquid cultures followed by washing to remove Tween 80 is time-consuming and can introduce inaccuracies. Two crucial observations in the development of this DST test may yield potential advantages in clinical application: (i) the φFN test performs well at (low) bacterial loads from 10^3^ to 10^5^ (**Figure 1C**), and (ii) when the standard concentration of Tween 80 in 7H9 was diluted 1000-fold, it did not inhibit the ability of the phage to infect *Mtb* (**Supplementary Figure S2**). Thus, the φFN-based workflow we have developed for DST of 7H9-cultured clinical isolates should be more efficient and convenient for operators to perform (**Figure 2C**). In this workflow, the isolates are merely 7H9-precultured to an OD_600_ over 0.2, which is indicated when the turbidity is visible by eye, and are used, irrespective of the degree of turbidity, diluted 1000-fold into the assay wells. Thus, although several clinical isolates may have different growth rates, they can be assayed in the same batch and do not need to be quantified by OD to provide the same bacterial loads. The resistance of *Mtb* to drugs was determined as RLR (referred to as RFU (Urdániz et al., 2016), RFR (Yu et al., 2016), RLU percentage (Rajagopalan et al., 2024) in previous studies).

The resistance of 71 of the isolates was identified by φFN DST assay, with a high degree of sensitivity and specificity (**Table 1**; **Figure 4**), providing compelling support for the general applicability of the φFN DST assay. In addition, the RLR values corresponded well with the level of drug resistance (**Figure 3D**), indicating the utility of RLR as an indicator of the degree of drug resistance of *Mtb* (Rajagopalan et al., 2024). However, because only a few samples were found which had RLR values in the 10% ~ 40% group, the relationship between RLR values and MICs for the different drugs could not be precisely determined and more *Mtb* with low level of drug resistance need to be tested. Though previous study showed no relationship between RLR vales and MICs for RIF, samples were not enough as well to make the conclusion. Thus, φFN DST assay exhibited high confidence in the detection of drug resistance for clinical isolated *Mtb* and might directly determine the level of drug resistance. In the future, with the help of φFN DST assay, it may be possible to detect the levels of drug resistance of *Mtb* within 3 days by simply dropping positive liquid culture of MGIT 960 into a well plate, regardless of the concentration of bacteria. However, the development of equipment for more automated testing is imperative and more clinical samples are required to provide additional validation.

In conclusion, we developed a new reporter phage, φFN, and a robust workflow for direct *Mtb* DST for four first-line antitubercular drugs by changing the concentration of *Mtb* and drugs, and found it performed well in clinical isolates compared with the golden standard MGIT 960 assay, but within 72 h only. Thus, the φFN DST assay will facilitate the direct detection of *Mtb* and drug resistance for controlling TB epidemics on a population-wide scale.

## Data Availability

The original contributions presented in the study are included in the article/**Supplementary Material**. Further inquiries can be directed to the corresponding authors.
