# Supplementary material for: Rapid detection of drug resistance in Mycobacterium tuberculosis clinical isolates for first-line antitubercular drugs by using a novel reporter mycobacteriophage
Source: Front Cell Infect Microbiol. 2025 Apr 10;15:1589236. doi: 10.3389/fcimb.2025.1589236 (PMC12018339; doi:10.3389/fcimb.2025.1589236)
Supplement: Supplementary file 1 [file DataSheet1.pdf]

Supplemental figures

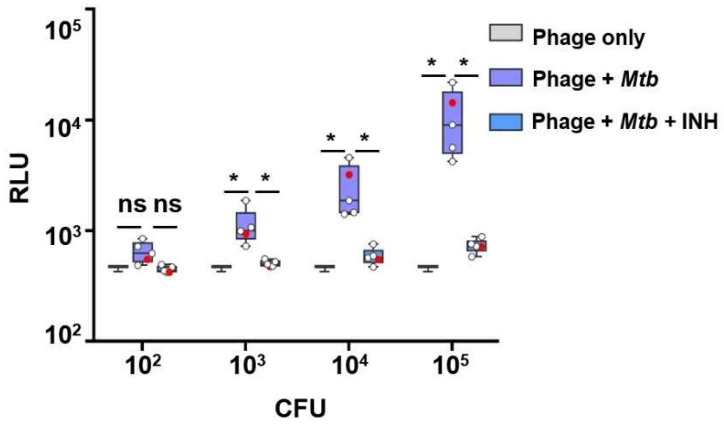

Fig. S1 Detection of INH resistance after 48-hour incubation with φFN. Ten-fold serial dilutions of four INH-sensitive clinical isolates and H37Rv were incubated for 48 hours with INH, then for a further 48 hours with φFN, in 96-well plates at 37°C before luminescence assay. Initial bacterial loads in wells were determined by spread plate on 7H11 agar. Red dots represented H37Rv. * represents $p < 0.05$, based on Student's *t*-test.

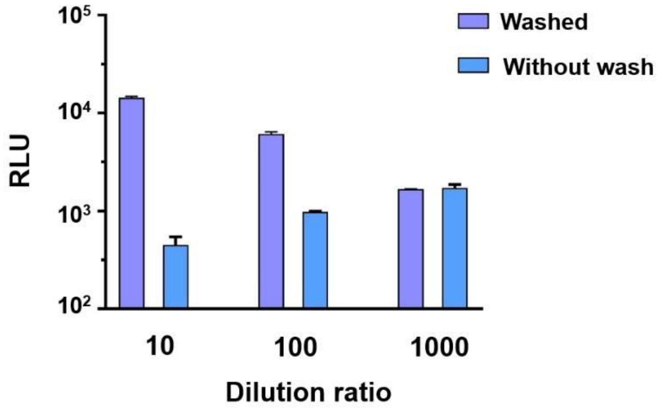

Fig. S2 Effect of Tween 80 concentration on the ability of φFN to infect *Mtb*.

Precultured H37Rv was either washed with detergent-free 7H9 medium or unwashed. Ten-fold serial dilutions were incubated with φFN for 24 h at 37°C before assay of RLU.
